# High-performance Fuel Cell with Stretched Catalyst-Coated Membrane: One-step Formation of Cracked Electrode

**DOI:** 10.1038/srep26503

**Published:** 2016-05-23

**Authors:** Sang Moon Kim, Chi-Yeong Ahn, Yong-Hun Cho, Sungjun Kim, Wonchan Hwang, Segeun Jang, Sungsoo Shin, Gunhee Lee, Yung-Eun Sung, Mansoo Choi

**Affiliations:** 1 Department of Mechanical Engineering, Incheon National University, Incheon, 406-772, Korea; 2 Global Frontier Center for Multiscale Energy Systems, Seoul National University, Seoul 151-744, Korea; 3 Center for Nanoparticle Research, Institute for Basic Science (IBS), Seoul 151-742, Korea; 4 School of Chemical and Biological Engineering, Seoul National University, Seoul 151-742, Korea; 5 Department of Chemical Engineering, Kangwon National University, Samcheok 245-711, Korea; 6 Division of WCU Multiscale Mechanical Design, Department of Mechanical and Aerospace Engineering, Seoul National University, Seoul, 151-742, Korea

## Abstract

We have achieved performance enhancement of polymer electrolyte membrane fuel cell (PEMFC) though crack generation on its electrodes. It is the first attempt to enhance the performance of PEMFC by using cracks which are generally considered as defects. The pre-defined, cracked electrode was generated by stretching a catalyst-coated Nafion membrane. With the strain-stress property of the membrane that is unique in the aspect of plastic deformation, membrane electrolyte assembly (MEA) was successfully incorporated into the fuel cell. Cracked electrodes with the variation of strain were investigated and electrochemically evaluated. Remarkably, mechanical stretching of catalyst-coated Nafion membrane led to a decrease in membrane resistance and an improvement in mass transport, which resulted in enhanced device performance.

Polymer electrolyte membrane fuel cell (PEMFC) has been extensively studied as an eco-friendly alternative energy device in the aspect of high energy conversion efficiency and continuous production of the electricity without pollutant emissions^1^,^2^,^3^,^4^. Recently, there have been several technical issues for the commercialization of PEMFC including water management at the cathode in the membrane electrode assembly (MEA)^5^,^6^,^7^,^8^ and resistance reduction of the electrolyte membrane^9^,^10^. To improve transport of water generated from electrochemical reaction at the cathode, research has been conducted on inserting meso/macro pore structures such as platinum inverse opal structure and pore formers into the electrode, which methods need chemical post-treatments^11^,^12^,^13^,^14^,^15^. Moreover, there have been attempts to reduce the resistance of an electrolyte membrane by lowering the thickness of the membrane. This thinning method, however, has remained as a challenge due to the inferior mechanical properties of the thinned membrane^2^,^10^.

Herein, in order to address these issues in a simple and effective way, we deliberately generated cracks on the electrode composed of carbon supported Pt particles (Pt/C) by stretching out a membrane electrode assembly (MEA) and used the stretched MEA in order to enhance mass transport in the electrode and to reduce ohmic resistance simultaneously. Generally, cracks are generated from the elastic mismatch between two attached surfaces with different elastic modulus when the surfaces are deformed^16^,^17^. Cracks have commonly been considered as defects^18^,^19^,^20^,^21^,^22^ and many studies have focused on how to avoid them. However, recent studies have indicated that cracks can be used to detect the strain force as in an ultrasensitive sensor^16^, microfluidic channels^17^ and so on. It is noteworthy that the crack can be a useful tool rather than a defect, when it is properly generated with a purpose of achieving a specific goal.

## Results

### Generation of cracks in the electrode by stretching out the catalysts-coated Nafion membrane

**Figure 1:**
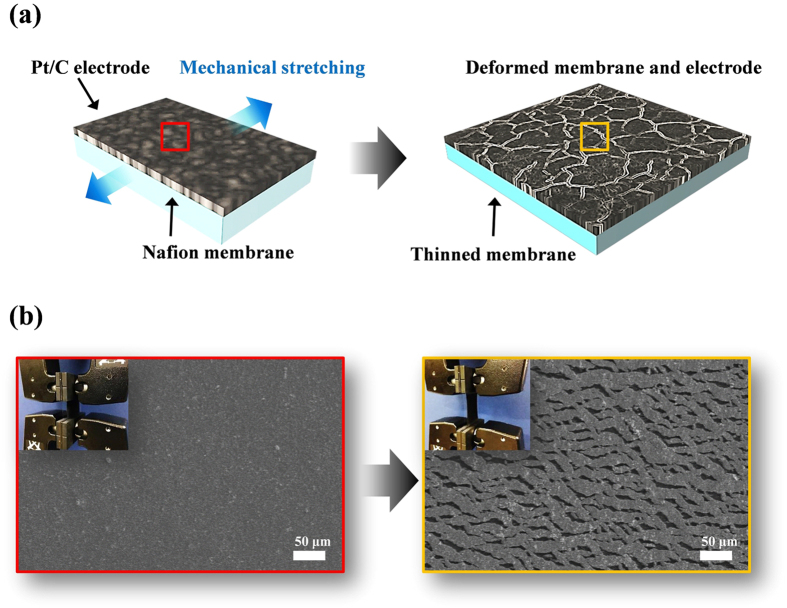
Generation of cracks in the electrode. (**a**) Schematic illustration of generating cracks in Pt/C catalyst layer with simply mechanical stretching. (**b**) Corresponding SEM images of catalyst layer before (left) and after (right) stretching out the catalyst coated membrane (Inset: corresponding optical images of stretcher machine).

Figure 1 shows the schematic illustration of crack generation in the electrode. A Nafion membrane has a relatively lower elastic modulus than a porous electrode with Pt/C. From this difference in elastic modulus called elastic mismatch, when the two attached surfaces are stretched out, cracks are generated on the porous electrode. If the Nafion membrane is stretched over the elastic deformation region, then it would not recover its original shape, and this means that the membrane has become longer than its original length due to the applied strain. And then, the cracks on the electrode also maintain their deformed shape along with the deformed membrane.

### Physical property of Nafion membrane

**Figure 2:**
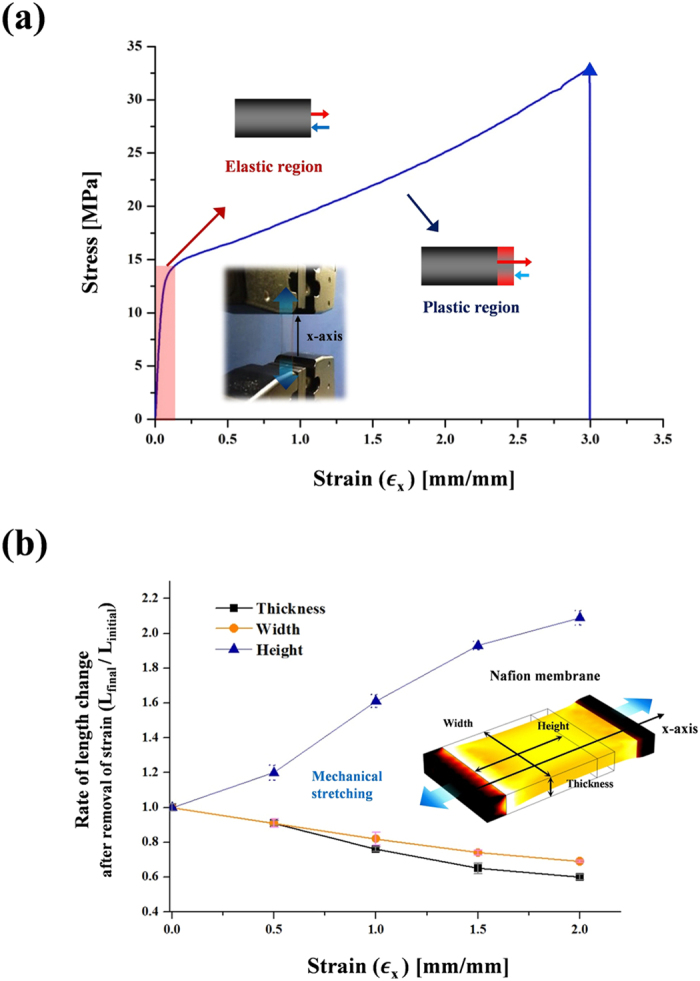
Physical property of Nafion membrane. Stretching properties of Nafion 212 membrane. (**a**) Strain-stress curves of Nafion membrane obtained by applying strain to the membrane until its breaks. (**b**) Changes of width, height and thickness of Nafion membrane with variation of strains (0.5, 1.0, 1.5 and 2.0).

To investigate the stretching property of a Nafion membrane, we conducted a strain test on a Nafion 212 membrane with a thickness of ~50 μm. As shown in Fig. 2, the membrane showed the maximum tensile strength of ~31.11 MPa and elongation of ~300% till its break. (Table S1) This result shows that the membrane can endure the stress until reaching the maximum tensile strength before it tears off, and can be stretched out to ~3 times of its original length, which is an intriguing feature in terms of stretchability. Furthermore, the shape of the strain-stress curve in Fig. 2a presents that the region of elastic deformation completely which has recovered to its original length is under ~0.08 strain, and it shows that the plastic deformation which has incompletely recovered in the region is above ~0.08 strain. The plastic deformation region is much wider than the elastic region and we used the plastic deformation of the membrane to simultaneously reduce the thickness of the membrane and generate cracks in the electrodes composed of Pt/C particles. As shown in Fig. 2b, we measured the rate of changes of width, height and thickness of a Nafion membrane according to the variation of strains (0.5, 1.0, 1.5 and 2.0) after removing them. And, we ascertained that changes of the membrane thickness were spatially uniform after the removal of strains as shown in Fig. S1.

### Morphological features of the generated cracks with variation of strains

**Figure 3:**
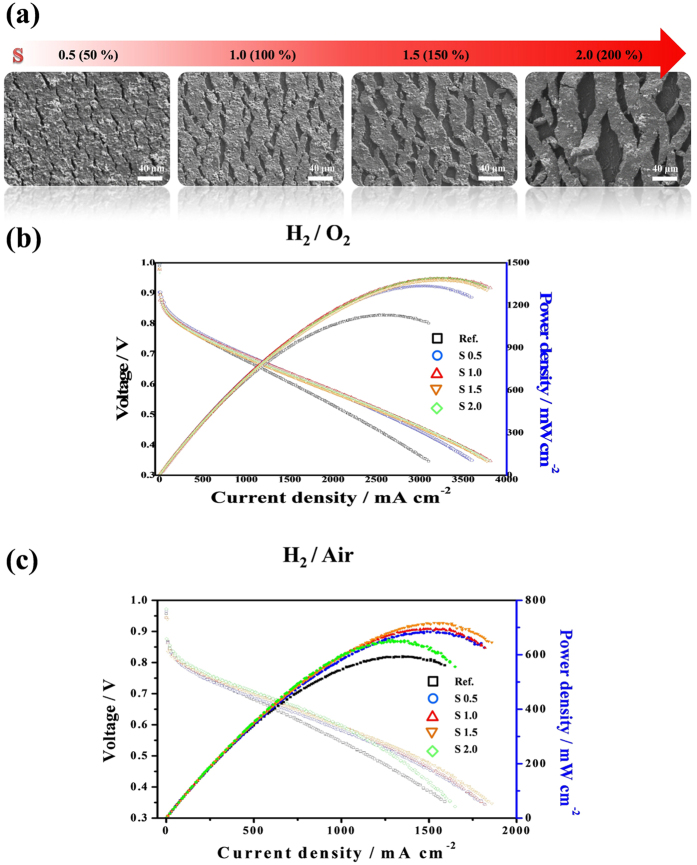
Morphological features of the generated cracks and measurements of device performance. (**a**) Corresponding SEM images of catalyst coated membrane after applying various strains (0.5, 1.0, 1.5 and 2.0). (**b**,**c**) Polarization curves of conventional membrane electrode assembly (MEA) and the MEA with electrode cracks with variation of strains (0.5, 1.0, 1.5 and 2.0) under the conditions of H_2_/O_2_ (b) and H_2_/Air (**c**).

To investigate the geometrical features of the cracks induced by applied strains, we observed the cracks with variation of strains by using a scanning electron microscopy (SEM) as shown in Fig. 3a. The catalyst-coated membranes were stretched out with variation of strains (S; 0.5, 1.0, 1.5, and 2.0) and areal fractions of the cracks with each strain in the corresponding SEM images were analyzed by using an image analysis program (ImageJ). As expected, the areal fractions of cracks increased as the value of strain increased. (~7.8% for S~0.5, ~13.4% for S~1.0, ~22.5% for S~1.5, and ~33.4% for S~2.0) Interestingly, we observed that the size of the cracks enlarged as the strain intensified. It implies that the cracks, or the macro-pores, in the catalyst layer can be controlled by the value of strain.

### Improved performance with cracked membrane electrode assembly (MEA) in polymer electrolyte membrane fuel cell (PEMFC)

To demonstrate the effect of cracks on the performance of PEMFC, we incorporated a cracked MEA into a single cell. This single cell with the cracked MEA was operated in a fully humidified condition of H_2_/O_2_ (H_2_/Air) and exhibited highly improved performance under all the conditions regardless of the strains compared with a conventional MEA, as shown in Fig. 3b,c. However, the MEA with a strain of ~2.0 applied exhibited more decreased performance than other samples under H_2_/air condition. This resulted from the catalysts debonded by high strain. When looking into the surface morphology of the stretched MEA at a lower magnification of the SEM (Fig. S2), we observed that only the catalyst layer of the stretched MEA with a strain of ~2.0 was detached from the membrane (In some cases, we observed with the naked eye that large Pt/C aggregates in the catalyst layer were locally separated from the membrane.). The detached catalyst layer would induce degradation of performance due to the increase of interfacial resistance and loss of the Pt catalyst. And, the result indicates that there exists the upper limit of applied strain to the catalyst-coated membrane and the optimal strain for the high performance of the MEA. In our experimental sets, the stretched MEA with 1.5 strain applied exhibited the highest performance. It showed the maximum power density of ~0.72 W cm^−2^ in the case of H_2_/air conditions, which power density is higher than the conventional one (~0.59 W cm^−2^) by ~22%. (Table S2) These performance enhancements can be explained by the effects from a crack acting as a macro-pore and the thinned membrane. First, the water transport enhancement which was confirmed by the difference of the power density that increased as the current density increased contributed to the performance enhancement of the MEA. Second, the membrane thickness reduced by stretching out has lowered ohmic resistance that occurs during the PEMFC operation. To address the stability issue, we conducted accelerated durability tests (ADTs) by repeated polarization test for ~1000 cycles with fully humidified H_2_/Air gases supplied to anode and cathode, respectively Even after ADT tests, the maximum power density of the stretched MEA was higher than the conventional MEA by ~36.7% in the case of H_2_/Air conditions under ambient pressure as shown in Fig. S3. Furthermore, the morphology of the cracks was maintained even after the durability test was carried out as shown in Fig. S4. There was no breakdown of the system even with the long term electrochemical stress. The results have come from the fact that pre-defined cracks from stretching out provide more available space that would distribute stress from the Nafion membrane when it swells and contracts. Hence, the stretched MEA with cracks have advantages in durability and long term stability than the conventional one.

### Electrochemical analysis of cracked MEA

**Figure 4:**
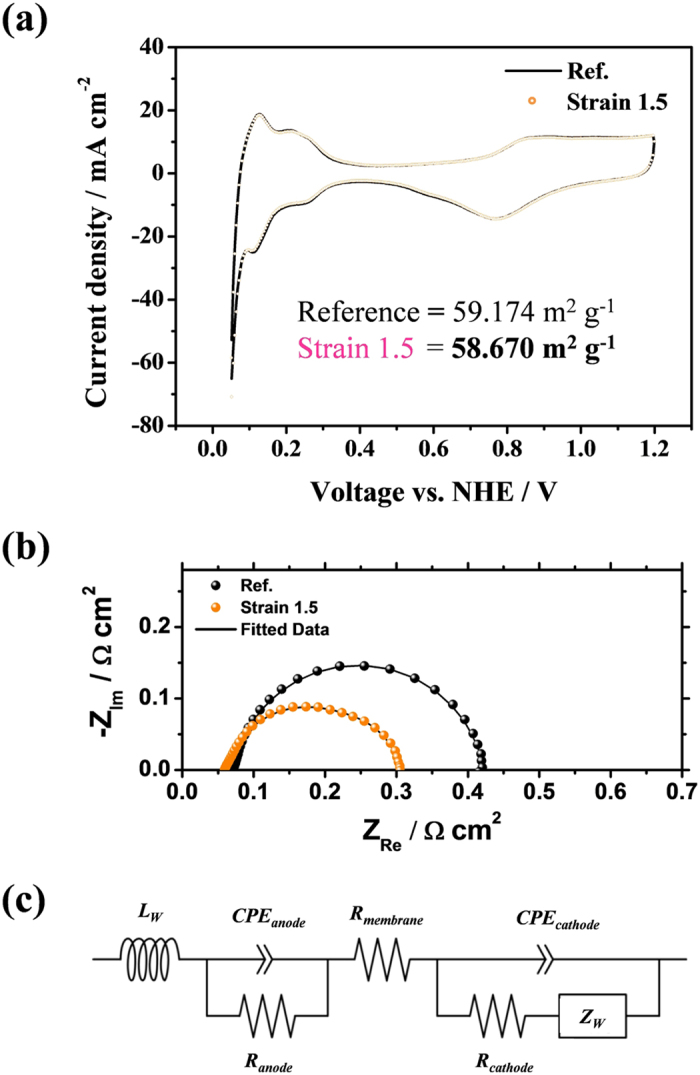
Electrochemical analysis. **(a)** Cyclic voltammogram (CV) of the cathode catalyst layers of a conventional MEA and the 1.5-strain applied MEA. **(b)** Electrochemical impedance spectroscopy (EIS) of a conventional MEA and the 1.5-strain applied MEA. at 0.6 V compared with RHE. (**c**) Equivalent circuit of the PEMFC (*L*_*W*_ = inductance of the electric wire, *R*_*membrane*_ = internal membrane resistance, *R*_*cathode (anode)*_ = charge transfer resistance of the cathode (anode), *CPE*_*cathode (anode)*_ = constant phase element of the cathode (anode) and *Z*_*W*_ = Warburg impedance).

To quantitatively investigate the effect of water transport enhancement and reduced ohmic resistance, both electrochemical impedance spectroscopy (EIS) and cyclic voltammetry (CV) were conducted (Fig. 4). As shown in Fig. 4a, comparable electrochemical active surface area (ECSA) was observed from the CV measurement^23^, which indicates that the generated crack doesn’t affect the area of tri-phase boundary during the operation. When the EIS data were fitted and calculated by the equivalent circuit (Fig. 4b)^24^, we found relatively lower ohmic resistance in the case of the 1.5-strain-applied MEA than that of the conventional one by ~14%, which resulted from the thinned membrane in the stretching process. Moreover, the Warburg impedance of the 1.5-strain-applied MEA (0.0393 Ω cm^2^) was much smaller than that of the conventional one (0.0691 Ω cm^2^), although the current density of the 1.5-strain-applied MEA was higher than that of the reference at 0.6 V. (Table S3) Furthermore, results of EIS measurement at the same current density (~1.4 A cm^−2^) show less resistance in the case of stretched MEA (S~1.5) than that of the reference as shown in Fig. S5. It means that the water removal from the cathode catalyst layer has been improved due to the existence of cracks. The enhanced water transport also can be confirmed by calculating the oxygen gain. The data of the oxygen gain were obtained by calculating potential difference when oxygen and air are supplied in the current density range and they indicate the degree of mass transfer in the cathode catalyst layer. In Fig. S6, the calculated oxygen gain of the 1.5-strain-applied MEA showed much lower values than the conventional one, which means that the generated cracks in the cathode catalyst layer removed the produced water much easily and helped the MEA to supply fuel gas more effectively than the conventional one.

## Discussion

To further investigate the mass transport effect while excluding the membrane thinning effect, we measured performances of the stretched MEA and the MEA with only the membrane stretched. The performance of the MEA with only the membrane stretched was slightly higher than that of the stretched MEA in the condition of H_2_/O_2_. However, in the condition of H_2_/Air, the stretched MEA showed much higher performance than the MEA with only the membrane stretched as shown in Fig. S7. In view of the commonly known fact that the performance in the condition of H_2_/Air is more affected by mass transport than that in the condition of H_2_/O_2_, the stretched MEA with cracks displayed positive effect in improving mass transport. We also conducted EIS measurement of the stretched MEA and the MEA with only the membrane stretched. Fig. S8a shows that they exhibited comparable ohmic resistances. The radius of the circle of the stretched MEA was much smaller than that of the MEA with only the membrane stretched, which implies that the generated crack has improved mass transport. Moreover, oxygen gain calculation for the experiments also supports our results (Fig. S8b).

In this study, we have presented, for the first time, a novel strategy to improve water transport and reduce ohmic resistance by simply stretching out the Pt/C-coated Nafion membrane. With the use of the unique stretching property of the Nafion membrane which has the maximum elongation of ~300% until its break, we have incorporated electrode cracks with the variations of strain (0.5, 1.0, 1.5 and 2.0) into a single cell. The generated cracks effectively enhanced water transport in the cathode catalyst layer, which was confirmed by oxygen gain and EIS measurements. Moreover, the thinned membrane from stretching out was found to reduce the ohmic resistance. These combinational effects of improved water transport and reduced ohmic resistance of the 1.5-strain-applied MEA resulted in performance enhancement compared by ~22% of the performance of the conventional one. Our novel approach of using cracks that have been considered as defects surely possesses potential to be applied to other energy conversion and storage devices. This technique can also be helpful, with its thinned MEA, in reducing the total volume of fuel cell stacks for compact applications such as in vehicles.

## Methods

### Preparation of membrane electrode assembly (MEA)

Catalyst ink was prepared by mixing water, 5 wt.% Nafion solution (DuPont) and isopropyl alcohol (IPA) (Aldrich) with the catalyst. 40 wt.% Pt/C (Johnson Matthey) was used for the cathode catalyst inks. The prepared catalyst ink was blended by ultrasonic treatment and sprayed onto the cathode side of bare Nafion 212 membrane to fabricate MEAs. The catalyst loadings were equally 0.2 mg cm^−2^ in the cathodes of the MEAs. These catalyst-coated membranes (CCMs) were dried at room temperature for more than 12 hours. After the process of stretching out the CCMs, the prepared catalyst ink was sprayed onto the anode side with 0.2 mg cm^−2^ catalyst loading. Then, gas diffusion layers (GDLs, SGL 35 BC), Teflon type gaskets and one channel serpentine-type (Fig. S9) were put onto the anode and cathode without a hot-press process.

### Process of stretching out prepared CCMs

As-prepared catalyst-coated membranes were stretched out by a stretcher machine (Intron Corp.), which can provide the strong binding force of the chuck and the uniform strain to the membrane. One side of the membrane was fixed and the other side of the membrane was pressed by two bite-blocks with the width of 5 cm. The initial length of the membrane was 2 cm and the membrane was stretched out with variation of strains (0.5, 1.0, 1.5, and 2.0). See detailed experimental process in Fig. S10. And the samples without the stretching process were also prepared as a reference.

### Physical analysis

Field emission-scanning electron microscopy (FE-SEM) was conducted using a SUPRA 55VP microscope (Carl Zeiss) to measure the morphology of the various samples used in this paper. The samples were observed at SE mode without additional coating processes.

### Electrochemical measurements

Prepared MEAs were assembled in a single cell (CNL PEM005-01, CNL Energy). For the single cell performance test at 70 °C, humidified H_2_ and O_2_ (air) gases were made to flow into the anode and cathode with the active geometric areas of 5.0 cm^−2^, respectively. The stoichiometric coefficient of H_2_/O_2_ (air) was 2.0/9.5 (2.0). Additionally, the relative humidity (RH) for the anode and cathode gases were 100%. To check repeatability, we tested five different samples which have the same applied strain. (Fig. S11). Electrochemical impedance spectroscopy (EIS) (Zennium, Zahner) of the single cells was measured at 0.6 V with an amplitude of 5 mV. The measurement was conducted in the frequency range from 0.1 Hz to 100 kHz. Other experimental conditions, such as temperature and gas humidification, were the same as the case for the single-cell operation at 70 °C with H_2_/Air. The ZView program (Scribner Associates Inc.) was used to fit the EIS data, and a simple equivalent circuit was applied as shown in Fig. 4c. Cyclic voltammograms (CVs) were obtained at 100 mV s^−1^ between 0.05 and 1.20 V to measure the electrochemical active surface (EAS) of the prepared cathode catalyst layers at room temperature. Humidified H_2_ and N_2_ gases were supplied to the anode and cathode, respectively, and the RH was 100% during the CVs measurement. The anode with H_2_ gas flowing around was used as the reference and counter electrodes, and the cathode with N_2_ gas served as a working electrode.

## Additional Information

**How to cite this article**: Kim, S. M. *et al.* High-performance Fuel Cell with Stretched Catalyst-Coated Membrane: One-step Formation of Cracked Electrode. *Sci. Rep.* **6**, 26503; doi: 10.1038/srep26503 (2016).

## Supplementary Material

Supplementary Information
