# Supplementary material for: High-performance Fuel Cell with Stretched Catalyst-Coated Membrane: One-step Formation of Cracked Electrode
Source: Sci Rep. 2016 May 23;6:26503. doi: 10.1038/srep26503 (PMC4876450; doi:10.1038/srep26503)
Supplement: Supplementary Information [file srep26503-s1.doc]

**Supplementary Information**

**High-performance Fuel Cell with Stretched Catalyst-Coated Membrane: One-step Formation of Cracked Electrode**

*Sang Moon Kim^1,2,3^*^†§^*, Chi-Yeong Ahn^4,5^*^†^*, Yong-Hun Cho^6^, Sungjun Kim^4,5^, Wonchan Hwang^4,5^, Segeun Jang^2,3^, Sungsoo Shin^2,3^, Gunhee Lee^2,3^, Yung-Eun Sung ^4,5*^ and Mansoo Choi ^2,3*^*

*^1^ Department of Mechanical Engineering, Incheon National University, Incheon, 406-772, Korea*

*^2^ Global Frontier Center for Multiscale Energy Systems, Seoul National University, Seoul 151-744, Korea*

*^3^ Division of WCU Multiscale Mechanical Design, Department of Mechanical and Aerospace Engineering, Seoul National University, Seoul, 151-742, Korea*

*^4^ Center for Nanoparticle Research, Institute for Basic Science (IBS), Seoul 151-742, Korea*

*^5^ School of Chemical and Biological Engineering, Seoul National University, Seoul 151-742, Korea*

*^6^ Department of Chemical Engineering, Kangwon National University, Samcheok 245-711, Korea*

^†^ These authors contributed equally to this work.

*Corresponding authors: mchoi@snu.ac.kr (M. Choi), ysung@snu.ac.kr (Y.-E. Sung)

**Figure S1.** Measurement of the membrane thickness after stretching out the membrane


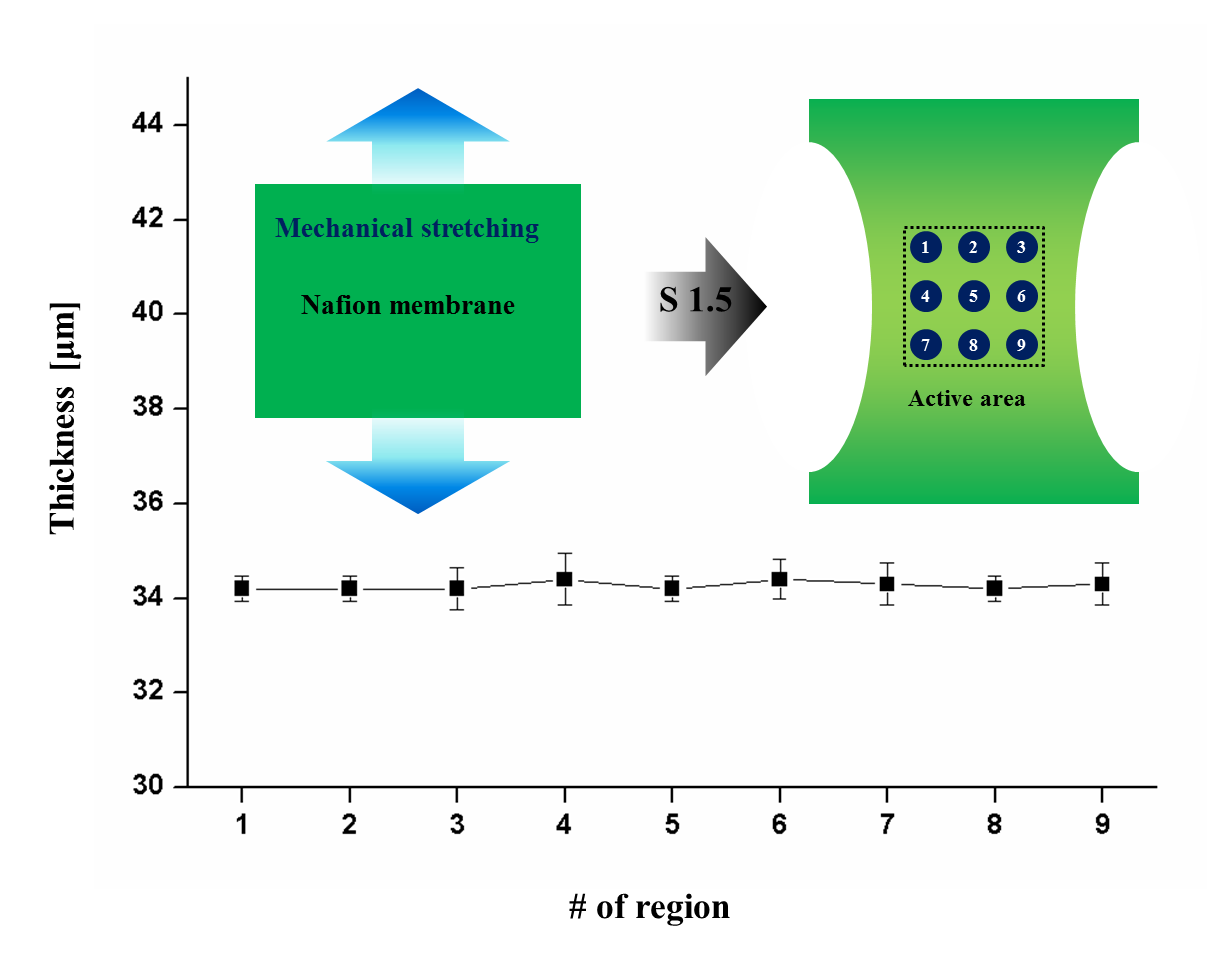


**Figure S2**. SEM images for catalyst layer of stretched MEA for each strain (0.5, 1.0, 1.5 and 2.0)


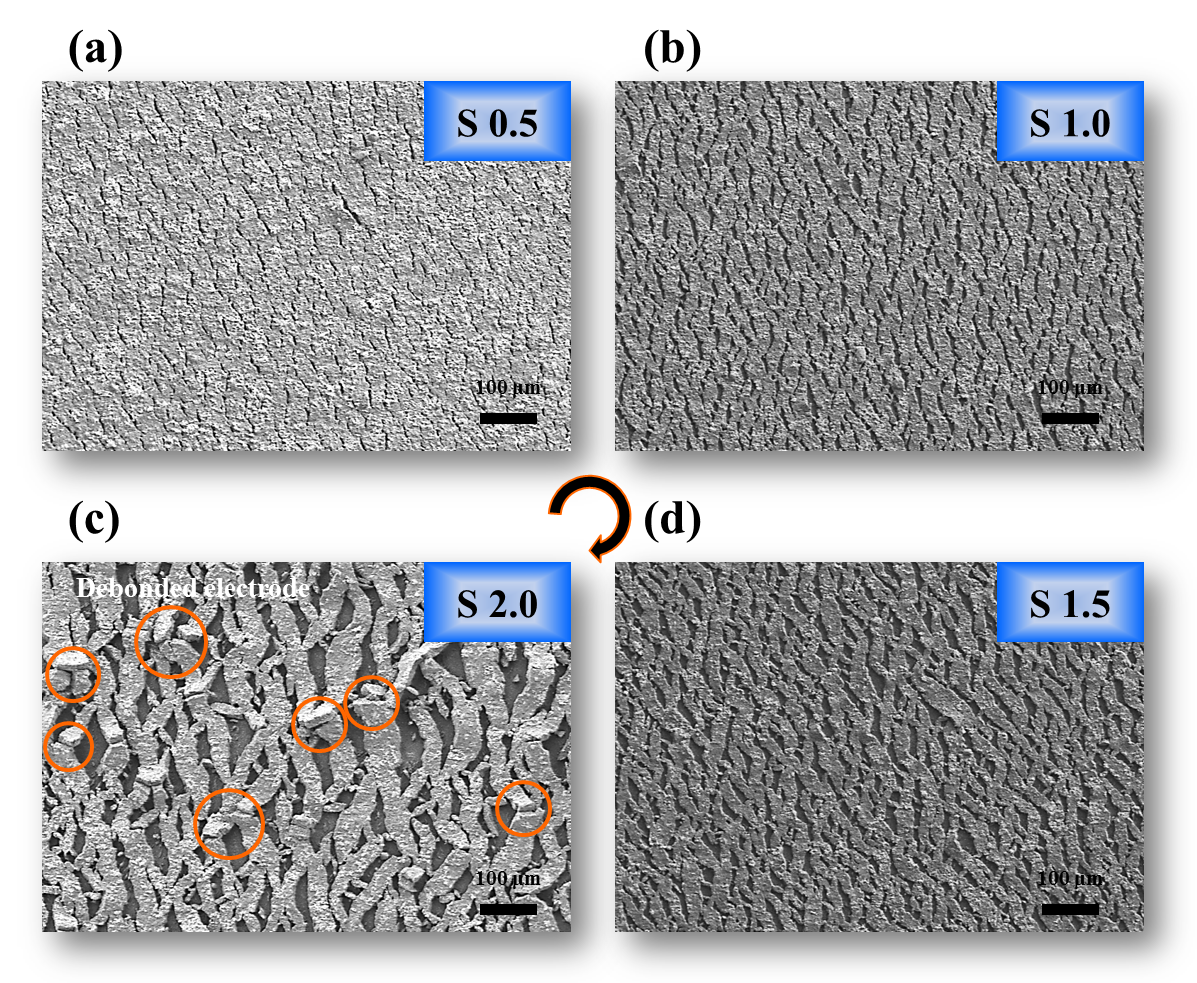


**Figure S3.** Accelerated durability test (ADT) for the stretched MEA with 1.5 strain and reference MEA


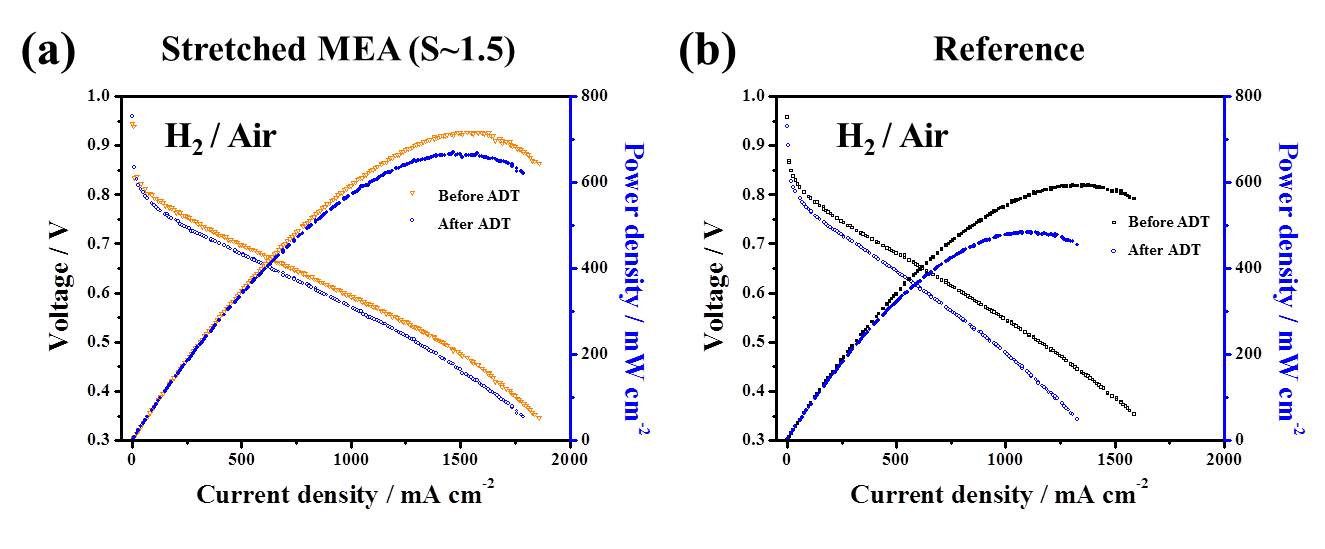


**Figure S4**. SEM images for catalyst layer of stretched MEA before ADT(a) and after ADT(b)


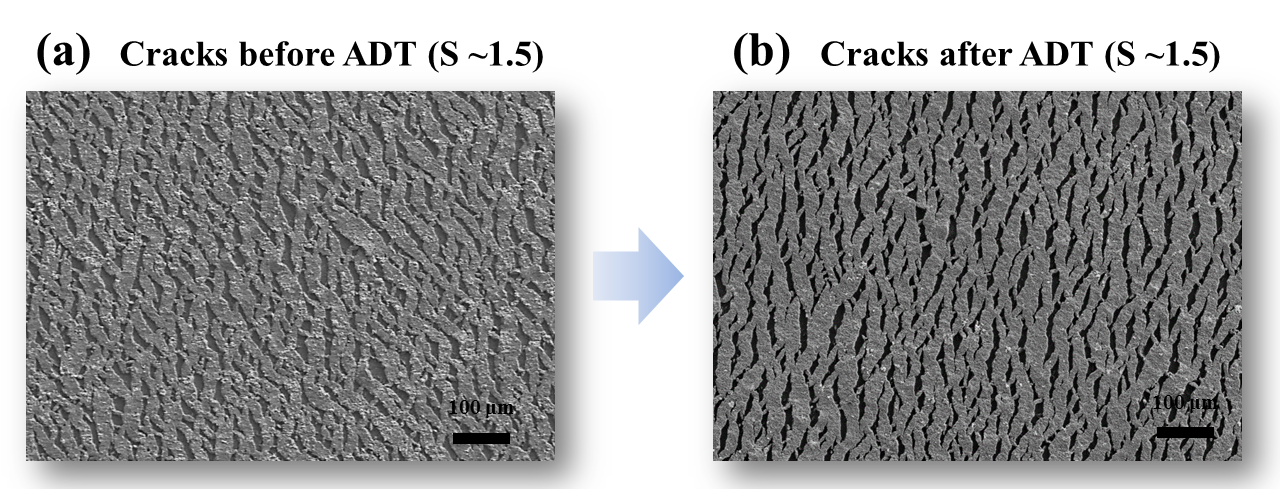


**Figure S5**. EIS measurements at the same current density (~ 1.4 A cm^-2^) for the sample of reference and stretched MEA (S ~1.5)


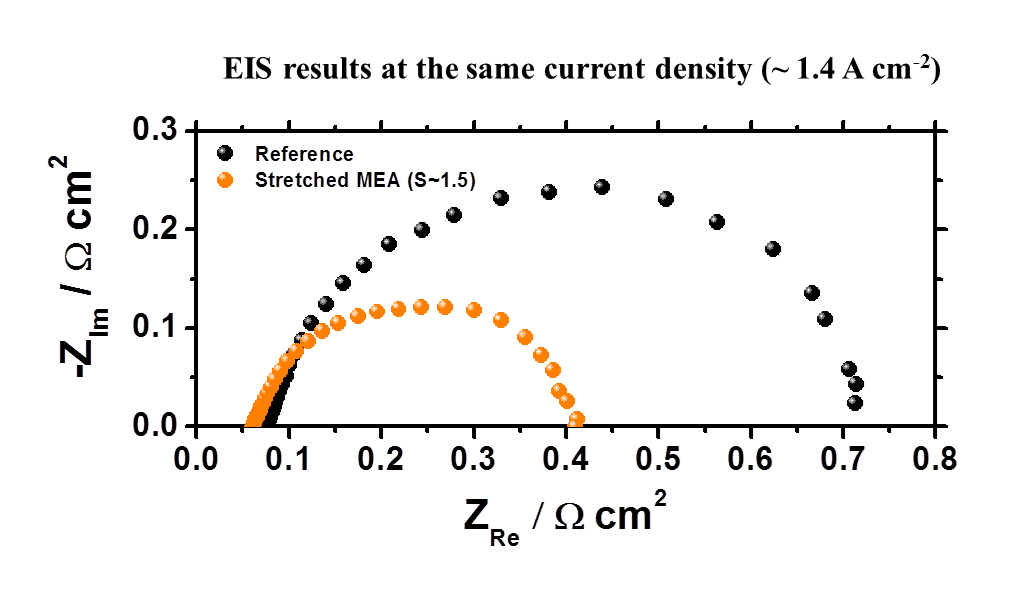


**Figure S6.** The graph for the oxygen gain obtained by calculating potential difference when oxygen and air are supplied respectively

**
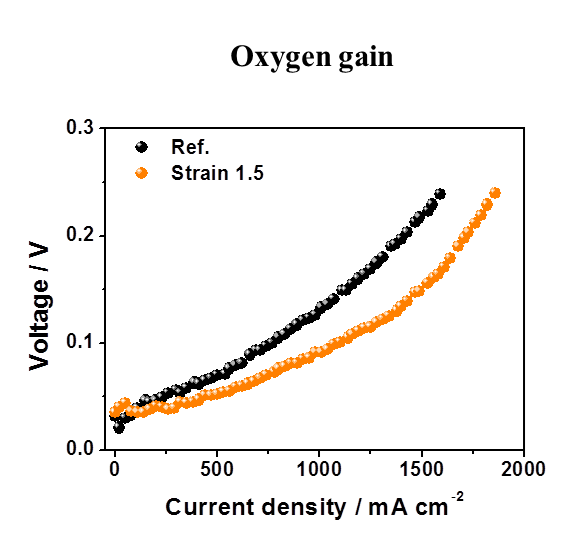
**

**Figure S7.** Polarization curves of the stretched MEA and the MEA with only membrane stretched. (a) H_2_/O_2_ condition, (b) H_2_/Air condition.


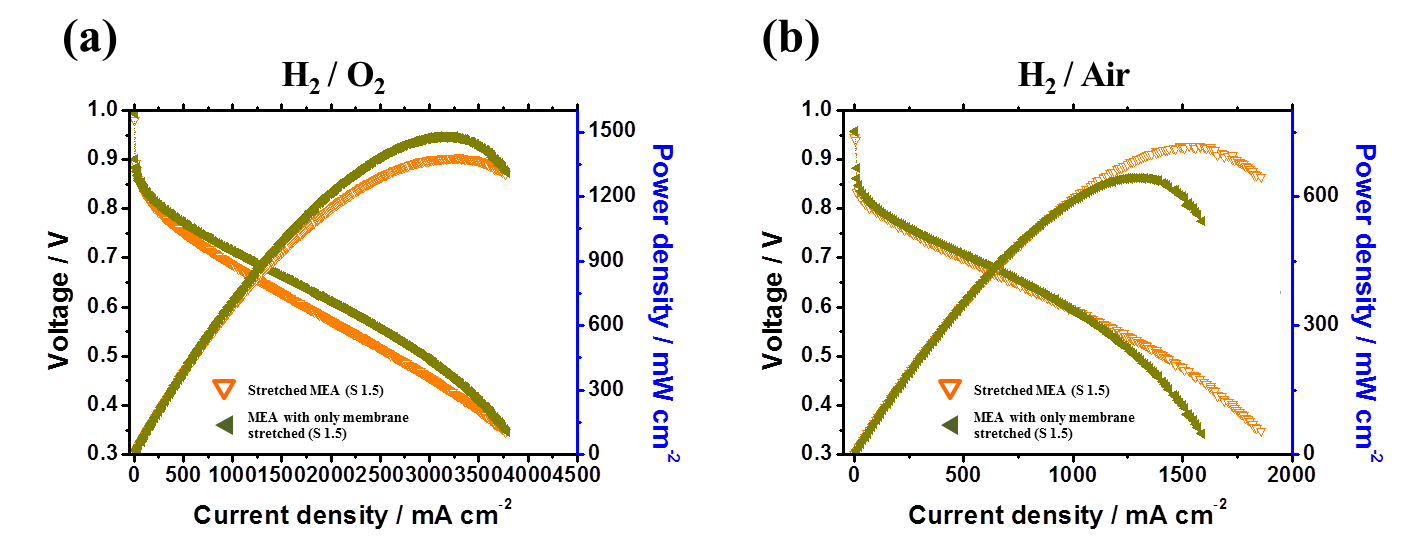


**Figure S8.** (a) EIS measurement of the stretched MEA and the MEA with only membrane stretched. (b) Oxygen gain calculation of the stretched MEA and the MEA with only membrane stretched.


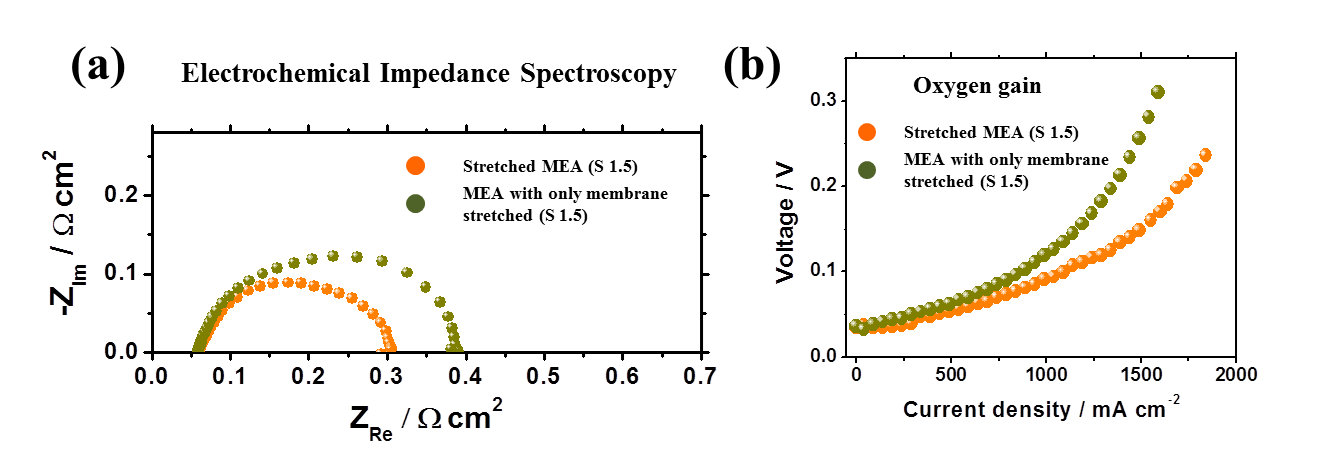


**Figure S9.** A camera image for one channel serpentine-type.


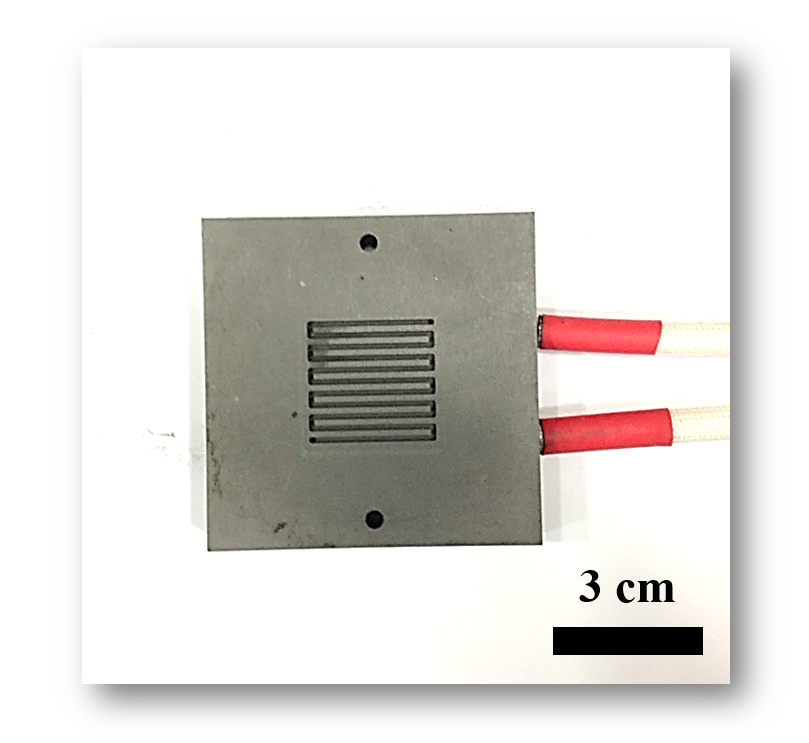


**Figure S10.** (a-c) Camera images for the preparation of stretched MEA within ~5 cm^2^ active area. (d) Changes of width and height of Nafion membrane with variation of strains (0.5, 1.0, 1.5 and 2.0).


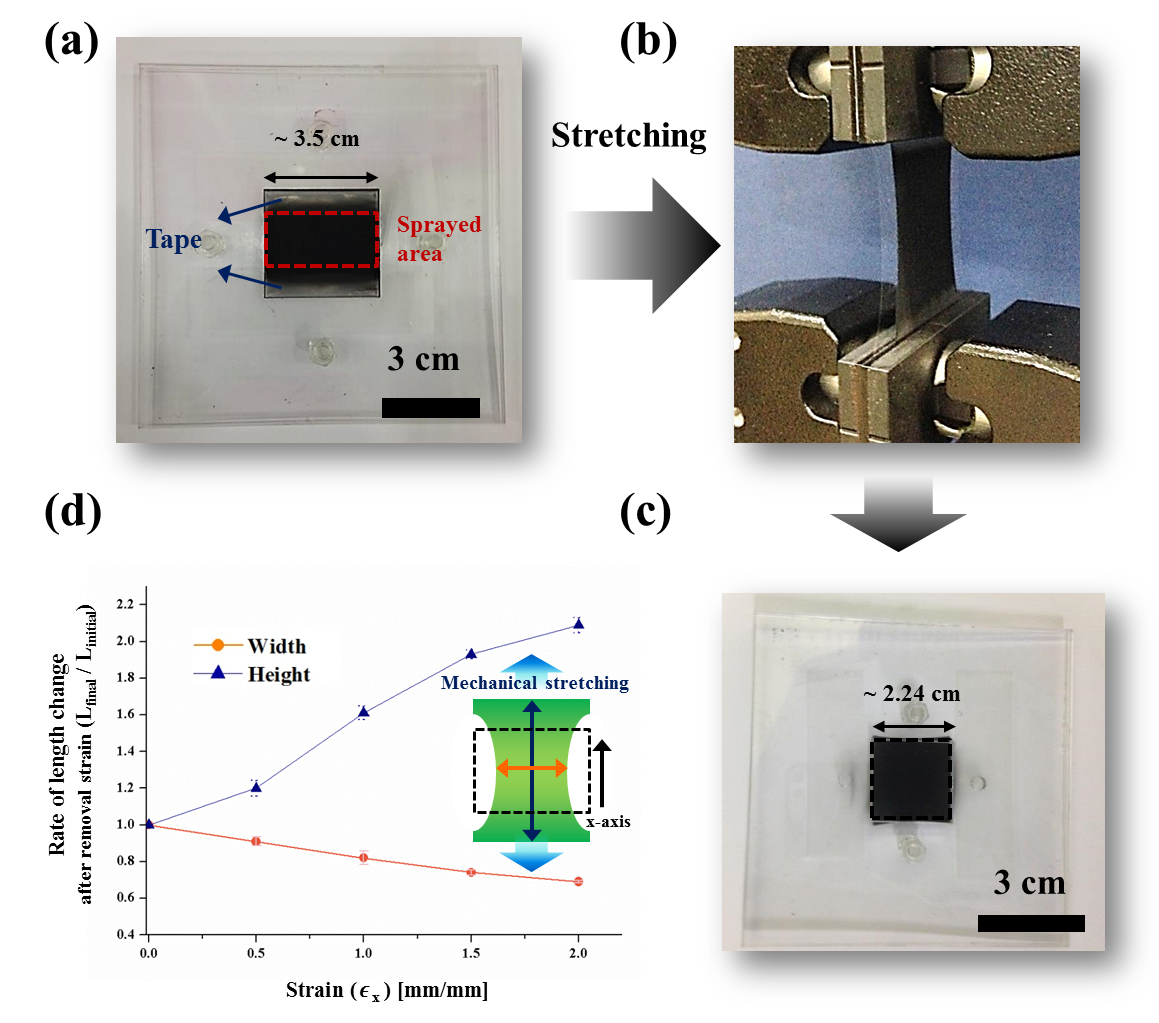


**Figure S11.** Polarization curves of five different samples for (a) the stretched MEAs (S ~1.5) and (b) conventional MEAs in the condition of H_2_/Air

**
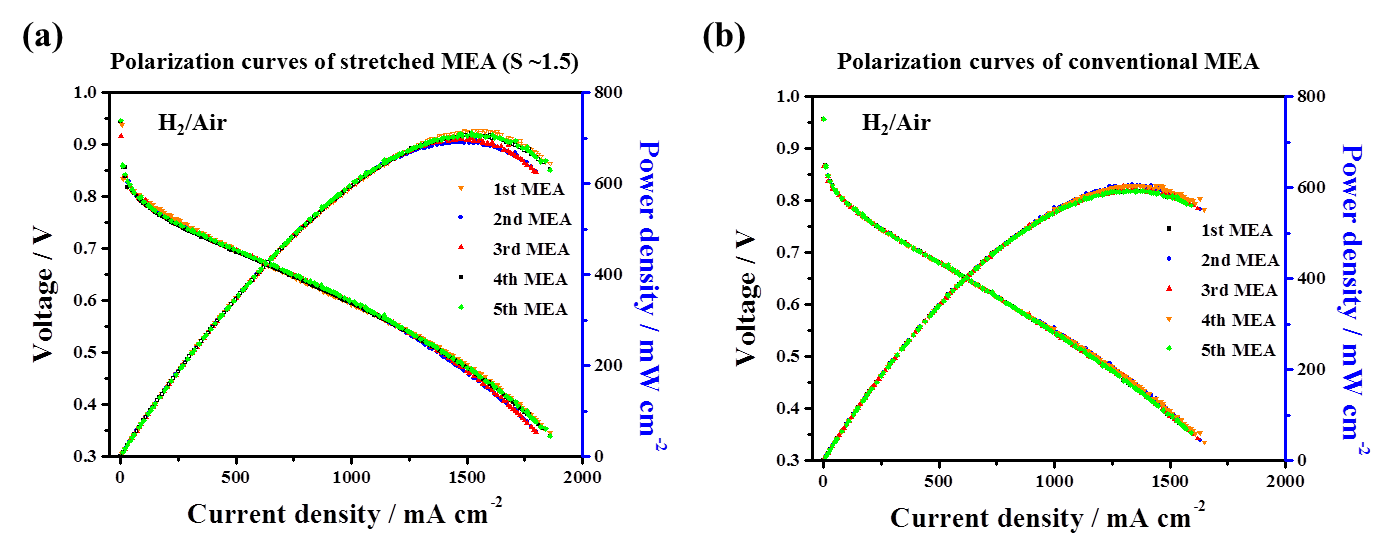
**

**Table S1.** Physical property of Nafion 212 membrane


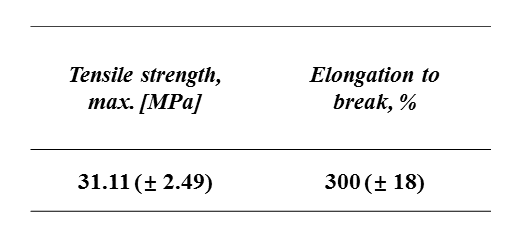


**Table S2.** Physical and electrochemical properties of the samples.


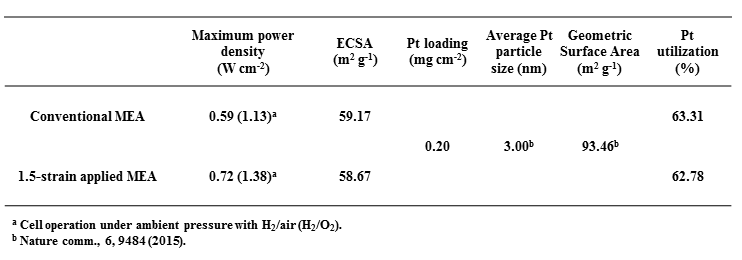


**Table S3.** Electrochemical impedance spectroscopy **(**EIS) fitted data.

**
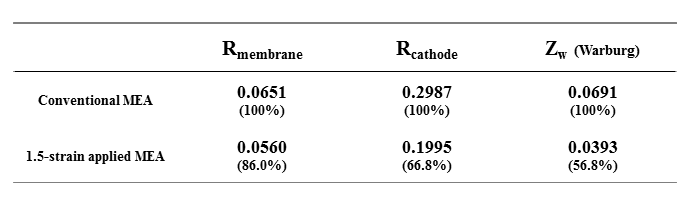
**
